# Mammals Preferred: Reassortment of *Batai* and *Bunyamwera orthobunyavirus* Occurs in Mammalian but Not Insect Cells

**DOI:** 10.3390/v13091702

**Published:** 2021-08-27

**Authors:** Anna Heitmann, Frederic Gusmag, Martin G. Rathjens, Maurice Maurer, Kati Frankze, Sabine Schicht, Stephanie Jansen, Jonas Schmidt-Chanasit, Klaus Jung, Stefanie C. Becker

**Affiliations:** 1 Department of Arbovirology, Bernhard Nocht Institute for Tropical Medicine, 20359 Hamburg, Germany; heitmann@bnitm.de (A.H.); mgr@froreich-bioscientia.de (M.G.R.); jansen@bni-hamburg.de (S.J.); jonassi@gmx.de (J.S.-C.); 2 Institute for Parasitology, Center for Infection Medicine, University of Veterinary Medicine Hannover, 30559 Hanover, Germany; Frederic.Gusmag@tiho-hannover.de (F.G.); Maurice.Andre.Maurer@tiho-hannover.de (M.M.); 3 Research Center for Emerging Infections and Zoonoses, University of Veterinary Medicine Hannover, 30559 Hanover, Germany; 4 Institute of Infectology, Friedrich-Loeffler-Institute, Federal Research Institute for Animal Health, Südufer 10, 17493 Greifswald, Germany; kati.franzke@fli.de; 5 Department of Pediatric Pneumology, Allergology and Neonatology, Hannover Medical School, Carl-Neuberg-Str. 1, 30625 Hanover, Germany; sabine.schicht@gmx.de; 6 Faculty of Mathematics, Informatics and Natural Sciences, University of Hamburg, 20148 Hamburg, Germany; 7 Institute for Animal Breeding and Genetics, University of Veterinary Medicine Hannover, 30559 Hanover, Germany; Klaus.Jung@tiho-hannover.de

**Keywords:** viral reassortment, orthobunyaviruses, insect cells

## Abstract

Reassortment is a viral genome-segment recomposition known for many viruses, including the orthobunyaviruses. The co-infection of a host cell with two viruses of the same serogroup, such as the *Bunyamwera orthobunyavirus* and the *Batai orthobunyavirus,* can give rise to novel viruses. One example is the Ngari virus, which has caused major outbreaks of human infections in Central Africa. This study aimed to investigate the potential for reassortment of *Bunyamwera orthobunyavirus* and the *Batai orthobunyavirus* during co-infection studies and the replication properties of the reassortants in different mammalian and insect cell lines. In the co-infection studies, a Ngari-like virus reassortant and a novel reassortant virus, the Batunya virus, arose in BHK-21 cells (*Mesocricetus auratus*). In contrast, no reassortment was observed in the examined insect cells from *Aedes aegypti* (Aag2) and *Aedes albopictus* (U4.4 and C6/36). The growth kinetic experiments show that both reassortants are replicated to higher titers in some mammalian cell lines than the parental viruses but show impaired growth in insect cell lines.

## 1. Introduction

With more than 88 different species, the *Orthobunyavirus* is the largest genus of arthropod-transmitted viruses worldwide (ITCV) [1]. Their amplification cycles involve vertebrate hosts and invertebrate vectors, and they are often associated with diseases in humans or livestock [1]. The genome of orthobunyaviruses consists of three negative-sense RNA segments (L, M and S) [1]. The S-segment encodes the non-structural protein S (NSs) and the nucleocapsid protein (N), and the L-segment encodes the RNA-dependent RNA polymerase L. Both L- and N-proteins are associated with the viral genome and form the ribonucleoprotein complexes (RNPs) [1]. The M-segment codes for the glycoproteins Gn and Gc and the non-structural protein NSm. The glycoproteins are embedded into the lipid bilayer enveloping the viral particles. The non-structural proteins NSs and NSm are involved in the interaction with the host and probably the vector’s immune system [1]. The genus *Orthobunyavirus* is classified into 18 serologically defined arbovirus serogroups [1]. All viruses of this genus have been classified in these groups based on complement-fixing antibodies’ serological relationships, hemagglutinating assay results and neutralising antibodies [2]. The Bunyamwera serogroup contains over 30 viruses, including *Bunyamwera orthobunyavirus* (BUNV) and *Batai orthobunyavirus* (BATV) [2]. BATV and BUNV infections in humans are primarily asymptomatic or are associated with very mild febrile symptoms [2]. BATV was initially isolated in 1955 from *Culex gelidus* mosquitoes in Malaysia [3] and subsequently described in several countries in Europe and Asia, including Germany [4,5,6]. BUNV was first discovered in Uganda [7] and is endemic in different sub-Saharan African countries and South America [8,9,10]. The above-described genome segmentation, close serological and genetic relationship and sympatric and simultaneous occurrence [11] of BATV and BUNV provide the basis for genetic reassortment. This process was first described for the *La Crosse orthobunyavirus* in mosquitoes [12] and subsequently for many different orthobunyaviruses [11]. Reassortment is especially frequent between viruses with a close genetic and antigenic relationship and is characterised by exchanging segments between both viruses in a co-infected cell [11]. A natural reassortant between BATV and BUNV is the Ngari virus (NRIV) [8,13,14], which was isolated from goats in Mauritania in 2010 [15]. NRIV carries the L- and S-segments of BUNV and the M-segment from BATV and has caused at least two outbreaks of infections in humans in Central Africa, e.g., in Kenya, Tanzania and Somalia between 1998 and 1999 and in Sudan ten years before. This virus shows increased pathogenicity compared to the parental viruses associated with hemorrhagic fever [13,16]. Besides reassortment, it is also possible that after co-infection, a four-segmented virus occurs. Using a reverse genetic system for Rift Valley Fever virus (RVFV), Wichgers et al. (2014) showed that bunyaviruses could pack four segments into a viral particle [17]. These four-segmented viruses were described as polyploid or diploid [18] and might be an intermediate step for reassortment [18]. 

This study aimed to analyse the capacity for reassortment of BATV and BUNV in different insect and mammalian cell lines. Furthermore, we characterised the replication of novel reassortant viruses in different cell lines to test for alteration of the viral replication due to reassortment, which might impact vector usage or pathogenesis in host animals. For this purpose, co-infections of BATV and BUNV in BHK-21 (*Mesocricetus auratus*), U4.4 and C6/36 (immune-competent and immune-deficient *Aedes albopictus* cells, respectively) were performed. The potential for reassortment in mammalian and insect cells was examined based on the frequency of isolated reassortants and diploid viruses. We developed segment-specific RT-PCRs for each virus to differentiate between the parental orthobunyaviruses and the reassortants.

## 2. Materials and Methods

### 2.1. Cells and Viruses

BHK-21 (*Mesocricetus auratus*; CCVL L 0179), Vero E6 (*Chlorocebus sabaeus*; CCVL L 0228) and PT (*Ovis aris*; CCVL L 0011) cells were cultivated in Minimum Essential Medium Eagle (MEM) supplemented with Hanks’ and Earle’s salts (Sigma-Aldrich, St. Louis, MO, USA), 10% fetal bovine serum (FBS), 1% non-essential amino acids and 1% sodium pyruvate solution (100 mM) (PAN Biotech GmbH, Aidenbach, Germany). SFT-R cells (*Ovis aris*; CCVL L 0043) were cultivated in MEM Eagle (Hank’s salts, Merck KGaA, Darmstadt, Germany) supplemented with 0.1 M sodium bicarbonate (Carl Roth, Karlsruhe, Germany) and 10% FBS. Huh7 cells (*Homo sapiens*; CCVL L 1079) were cultivated in Gibco™ Ham’s F-12 (Thermo Fischer Scientific, Waltham, MA, USA), with 10% FBS, Iscove’s Modified Dulbecco’s Medium (Thermo Fischer Scientific) and 0.1 M sodium bicarbonate (Carl Roth, Karlsruhe, Germany). In addition, 1% penicillin/streptomycin was added to all culture media. All mammalian cells were derived from the cell bank of the Friedrich-Loeffler-Institute (Friedrich-Loeffler-Institute, Riems, Germany) and cultivated at 37 °C with 5% CO_2_. FBS concentration was reduced to 5% for virus maintenance, plaque assays, TCID_50_, plaque-purification and co-infection experiments.

U4.4 (*Aedes albopictus*, CVCL_Z820) (kindly provided by Sandra Junglen, Institute for Virology, Charité, Berlin, Germany), C6/36 (*Aedes albopictus*; CVCL_Z230) (Friedrich-Loeffler-Institute, Riems, Germany) and Aag2 cells (*Aedes aegypti*; CVCL_Z617) (kindly provided by Alain Kohl, MCR-University of Glasgow Center for Virus Research, Scotland, UK) were cultivated in Schneiders’ Drosophila Medium (PAN Biotech GmbH, Aidenbach, Germany) supplemented with 10% FBS (gold(PAN Biotech GmbH, Aidenbach, Germany)), 1% l-glutamine solution (200 mM), 1% MEM NEAA (100×) without L-glutamine, 1% sodium pyruvate solution (100 mM) and 1% penicillin/streptomycin (PAN Biotech GmbH, Aidenbach, Germany—P06-07050) at 25 °C.

BUNV ATCC^®^ VR-87 [7] and BATV Strain 53.2 [6] were grown alternating on either BHK-21 cells or C6/36 cells. BHK-21 cells were seeded in T25 cm^2^ tissue culture flasks (Sarstedt AG & Co., Nümbrecht, Germany) at a density of 2 × 10^6^ cells per flask infected at a multiplicity of infection (MOI) of 0.01 PFU per cell. The supernatant was harvested five days post-infection (p.i.). C6/36 cells were seeded at 5 × 10^6^ cells per T25 cm^2^ flask and infected at an MOI of 0.1. The supernatant was harvested seven days p.i.. Viruses were concentrated using 5× Peg-it™ Virus Precipitation Solution (System Biosciences, LLC, Palo Alto, CA, USA, Cat. # LV810A-1) following manufacturer protocol. Viral titers were determined using TCID_50_ and plaque assay. Viruses were stored at −80 °C.

Plaque assays were performed in 24-well plates (Sarstedt AG & Co., Nümbrecht, Germany) using 2 × 10^6^ Vero E6 cells per plate. One day post-seeding, each well was incubated with 100 µL diluted viral supernatant (10^−2^–10^−7^) for 60 min at 37 °C and 5% CO_2_. Afterwards, supernatants were discarded, cells were washed with 1× PBS and layered with 1% methylcellulose solution. For staining, cells were fixed 4 to 5 days p.i. with 4% formalin solution for 30 min, washed with 1× PBS and covered with crystal-violet (10% crystal-violet and 3,7% (*v*/*v*) 37% formaldehyde) for at least 30 min or overnight.

TCID_50_ were performed in 96-well tissue culture plates (Sarstedt AG & Co., Nümbrecht, Germany) using BHK-21 cells. Cells were grown in T25 cm^2^ tissue culture flasks until confluency was reached, detached and split 1:4 in Dulbecco’s Modified Eagle’s Medium (DMEM), supplemented with 5% FBS and 1% sodium pyruvate solution (100 mM) (=ready-to-use DMEM). The cell solution was divided into four 96-well tissue culture plates (100 µL/well). Each well was supplemented with 99 µL DMEM and 11 µL/well virus-dilution (10^−1^–10^−8^). Plates were incubated at 37 °C and 5% CO_2_ until cytopathic effects (CPE) were visible. Calculations were performed according to Reed and Muench [19] and converted to PFU/mL (PFU/mL = 0.69 × TCID_50_/_mL_).

### 2.2. Co-Infection

For co-infections in BHK-21 cells, 2 × 10^6^ cells per well were seeded in 6-well plates, infected at an MOI of 5 (BATV: BUNV ratio 1:1) or at an MOI of 5 and 0.1 (BATV: BUNV ratio 50:1) and incubated at 37 °C with 5% CO_2_. Co-infections in U4.4 and C6/36 cells were performed with 5 × 10^6^ cells per 6-well plate, infected at an MOI of 1 (BATV: BUNV ratio 1:1) and incubated at 25 °C. Three days p.i. the supernatants of all co-infections were harvested, and viral titers determined via plaque assay.

### 2.3. Plaque Purification

Vero E6 cells were seeded in 6-well plates with a density of 2 × 10^6^ cells/well. Cells were infected with viral supernatants from co-infection experiments diluted to approx. 50 PFU per well. Plates were incubated at 37 °C and 5% CO_2_. After 60 min, supernatants were discarded, cells were washed with 1× PBS and layered with 1% LMP agarose solution. For the LMP agarose solution, UltraPure^TM^ LMP Agarose (Amresco, Solon, OH, USA) was solubilised in sterile water, chilled to 37 °C and supplemented with pre-heated, ready-to-use DMEM. After the LMP agarose hardened at room temperature for 60 min, the plates were incubated for at least five days at 37 °C and 5% CO_2_. Depending on the plaque size, the incubation time was increased to 7 days. Single plaques were picked with a 10 µL pipet tip and cultivated on 2 × 10^6^ BHK-21 cells/well in 24-well plates at 37 °C and 5% CO_2_.

### 2.4. RNA Extraction, RT-PCR and NGS

To examine the genome composition of virus isolates derived from co-infection experiments, 140 µL of each plaque-purified co-infection isolate was inactivated in AVL buffer (QIAamp^®^ Viral RNA Mini Kit, Qiagen, Hilden, Germany). According to the QIAamp^®^ Viral RNA Mini Kit protocol, subsequent RNA extractions were performed. Segment-specific RT-PCRs for BATV and BUNV were performed with the QIAGEN^®^ One-Step RT-PCR Kit (Qiagen, Hilden, Germany) in 20 µL reactions with 8 µM forward and a reverse primer each (Supplementary Table S1) and 2 µL RNA template. After the reverse transcription (50 °C for 30 min) and an initial denaturation step (95 °C for 15 min), a 35 cycles 3-step amplification was performed with 1 min denaturation (95 °C), 30-sec annealing at 50 °C and 2 min elongation (72 °C). PCR results were visualised on a 1% TAE-agarose gel.

Next-generation sequencing (NGS) and sequence alignments were performed at the Institute for Diagnostic Virology of the Friedrich-Loeffler Institute (Riems, Germany) by the group of Dr. Dirk Höper as previously described [20]. Isolated RNA was transcribed into double-stranded cDNA (cDNA Synthesis System Kit, Roche, Mannheim, Germany), fragmented via ultrasonication (M220 Focused-ultrasonicator, Covaris, Woburn, MA, USA) and subsequently used for library preparation (SPRI-TE instrument, Beckman Coulter, Krefeld, Germany). Quantification of the resulting libraries was performed with KAPA Library Quant Illumina/Universal Kit (KAOA biosciences, Cape Town, South Africa) and final sequencing with a MiSeq instrument (Illumina, San Diego, CA, USA) and the Illumina MiSeq Reagent Kit v3 (600 cycle, Illumina).

### 2.5. Growth Kinetic Experiments

Viral growth experiments were performed in 24-well plates. With 1 × 10^5^ cells per well, BHK-21, HuH7, PT and SFT-R cells were first incubated in a 50-mL tube as a suspension with an MOI of 0.1 for 60 min at 37 °C with 5% CO_2_. Cells were centrifuged at 500× *g* for 5 min, washed with 1× PBS (three times) and finally resuspended in ready-to-use DMEM with FBS concentration reduced to 5% and seeded in a 24-well plate. Growth kinetics in U4.4, C6/36 and Aag2 cells were performed with 1 × 10^6^ cells/well at an MOI of 0.1. Incubation was at room temperature for 60 min. Afterwards, cells were washed with 1× PBS and resuspended with ready-to-use Schneiders’ Drosophila Medium before seeding. Incubation was at 25 °C, and at different time points, the supernatant was stored from one well for each experiment (0, 24, 48 and 72 h post-infection, 48 h p.i only for mammalian cells).

### 2.6. Statistical Methods

Fractions of clones from co-infection experiments were compared using Fisher’s exact test. Growth kinetics were analysed using Student’s t-test and ANOVA. Statistical analyses were performed with GraphPad Prism v9 (San Diego, CA, USA).

Raw values were first normalised for all growth kinetics to eliminate measured virus contents at time point zero. Therefore, the mean value for each virus at time point zero was calculated, and all values for this virus were divided by this mean. Next, these ratios were log-transformed for variance stabilisation and to bring values closer to a normal distribution. This step is necessary because measurements at the later time point have larger means and larger variances. Without variance stabilisation, time points would not be comparable. Finally, log-transformed ratios were shifted such that each time curve had a start value of zero.

After normalisation, time curves in each cell line were analysed by a two-way analysis of variance, including the effect of the four viruses, the four time points and the interaction between viruses and time. Studying the interaction is of particular interest to detect whether time profiles of individual viruses are parallel. An analysis of variance was performed using the lme3- and lmer test packages for the statistics software R (version 4.0.4, www.r-project.org (accessed on 1 February 2021)) to account for the repeated measurements. Subsequent comparisons with reduced models, including one “real” and one “combined” virus, were performed for those cell lines where the interaction p-value of the full model was close to significance, i.e., reduced models refer to models applied to a subset of the data. *P*-values in the full model were considered significant at a level of 5%, and *p*-values in the four reduced models at a Bonferroni-corrected level of 5%/4.

For visualisation, time-specific 95% confidence intervals for each virus of the form “mean +/− z1-α/2 * standard error of the mean” were calculated, where z1-α/2 denotes the 1-α/2-quantile of the standard normal distribution.

## 3. Results

### 3.1. Development of Segment-Specific RT-PCR

**Figure 1:**
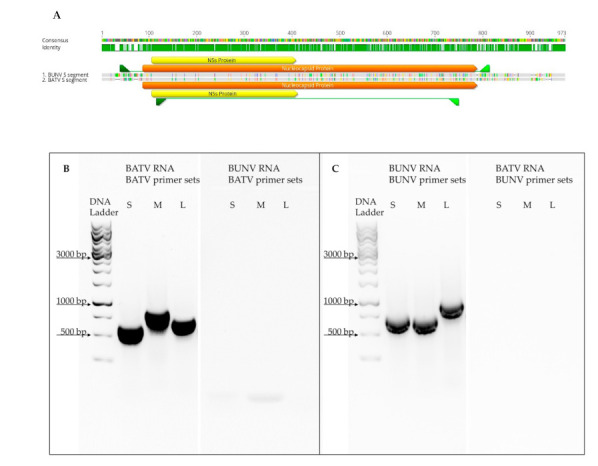
(**A**) Sequence alignment of BATV and BUNV S segments and the primer’s respective placement for unique amplification. Visualisation with Geneious Prime 2021.0.3. (**B**,**C**) Representative agarose gel runs after amplifying BATV and BUNV L-, M- and S-segments using segment-specific primer sets (Supplementary Table S1). (**B**) BATV primer sets; (**C**) BUNV primer sets.

We developed segment-specific RT-PCR primer sets to enable an unambiguous classification of BATV, BUNV and arising reassortants. We identified virus-specific regions for each segment based on sequence alignments of the BATV and BUNV strains used for co-infection. These unique regions were then used to design primer sets as exemplified for the S-segment shown in Figure 1A. We evaluated our primer sets’ specificities with RNA preparations of BATV and BUNV (Figure 1B,C). RT-PCR with each primer set resulted in a specific amplification product, allowing the examination of reassortment events between BUNV and BATV.

### 3.2. Reassortment during Co-Infection in Mammalian and Insect Cells

**Figure 2:**
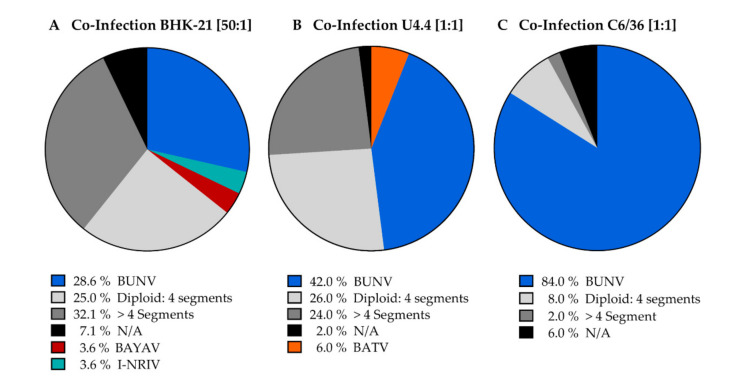
The fraction of total results from co-infection experiments. (**A**) Displays the results from co-infection of BHK-21 cells with *Batai orthobunyavirus* (BATV) and *Bunyamwera orthobunyavirus* (BUNV), (*n* = 28). Single plaque isolation revealed two direct reassortment events. One isolate, named I*-*NRIV, is a BUNV reassortant that integrated the BATV M-segment. The second isolate was named BAYAV; it is a BATV reassortant that integrated the BUNV L-segment. (**B**) Shows the results for co-infection in U4.4 insect cells (*n* = 50). (**C**) Shows the results after co-infecting C6/36 cells (*n* = 50). Isolates that were RT-PCR positive for more than four segments are given as >4 segments, and N/A specifies samples where detection by RT-PCR failed.

To assess the potential for reassortment of BUNV and BATV in mammalian cells, we co-infected BHK-21 cells at an MOI of 5. Out of each co-infection, we plaque-purified 50 isolates and analysed them via RT-PCR as described above. After the initial plaque purification, 34 isolates (68%) had a BUNV wild-type (wt) genome (Supplementary Figure S1A). None of the isolates were BATV wt. All diploid isolates were BUNV that incorporated BATV segments into the virion (24%) in which the diploid segments’ frequency was negatively correlated with the segment size was (Supplementary Figure S1B). Nine isolates were diploid for the S-segment, two isolates for the M-segment and only one isolate was diploid for the L-segment. We could not observe segment reassortment after co-infection. However, after a second plaque-purification, although most diploid viruses showed a reversion to BUNV wt (Supplementary Figure S1C), we observed one transition of a diploid virus to a reassortant virus (Supplementary Figure S1D). Next, we tested if the reassortment efficiency can be enhanced by reducing the MOI of BUNV to 0.1 while keeping the initial infection dose for BATV at MOI 5 (50:1 BATV:BUNV infection). After plaque-purification of 28 isolates, only 28,6% (*n* = 8) showed BUNV wt segment composition (Figure 2A). Fischer’s exact test revealed a significant reduction of BUNV isolates in the 50:1 infection (*p* = 0.001). The proportion of diploid isolates increased to 25% (*n* = 7). Once more, all diploid isolates were of BUNV origin. Six isolates were diploid for the S-segment and one isolate diploid for the L-segment. Again, the BATV wt virus was not observed. Two reassortant viruses were isolated (7.2%). The second plaque-purification revealed that both segment combinations were stable for at least one passage and replicated in BHK-21 and Vero E6 cells. The first reassortant virus consisted of the same segments as the already known NRIV, namely BUNV L- and S-segments and the BATV M-segment. Since the reassortant virus had the same segment composition, but a different genomic sequence due to a German BATV isolate used for co-infection, we named it Ngari-like virus (I-NRIV). Interestingly, the second reassortant virus incorporated the BUNV L-segment into the BATV genome (BUNV L, BATV M and S). We provisionally named this new reassortant Batunya virus (BAYAV). The I-NRIV genome was sequenced along with BATV and BUNV, and sequence alignments confirmed the segment reassortment. No mutations were found compared to the parental virus isolates (sequences available via GenBank # MZ773502, MZ773503, MZ773504, MZ773505, MZ773506, MZ773507, MZ773508, MZ773509, MZ773510).

Next, we analysed the potential for reassortment in the insect cells using U4.4 (Figure 2B) and C6/36 (Figure 2C) cells, which both can be infected with BATV and BUNV [12]. After the co-infection of cells with an MOI of 1 for both viruses (1:1 ratio), we plaque-purified 50 isolates from each co-infection and analysed their genomic composition. Compared to the 1:1 ratio in BHK-21 cells, less BUNV isolates were found (*n* = 21, 42%). Furthermore, we found 22% of all isolates were BUNV with an additional BATV segment (*n* = 11), 4% BATV with additional BUNV (*n* = 2) and 6% (*n* = 3) were BATV wt (Figure 2B). We did not detect reassortant viruses. A second plaque-purification of a diploid isolate resulted in the genome’s reversion back to three segments, with 100% re-isolation of BUNV wt virus (data not shown). To evaluate the role of a functional RNAi response during viral reassortment, we co-infected immune-deficient C6/36 cells lacking a functional Dicer-2 enzyme [21], resulting in an impaired antiviral immune response. The number of BUNV isolates was strikingly increased in these cells with 84% (*n* = 42) (Figure 2C) of all plaque isolates having a BUNV wt genome; Fisher’s exact test revealed a highly significant difference (*p* < 0.0001) in comparison to U4.4 cells. In contrast, no BATV wt genome and only four diploid viruses (8%) were detected. All diploid isolates were BUNV that incorporated BATV S into the virion. Fisher’s exact test showed a significant reduction of diploid viruses compared to U4.4 cells (*p* = 0.031). Similar to the co-infection in U4.4 cells, no reassortant viruses occurred. A second plaque-purification of a diploid virus resulted in the genome’s reversion back to BUNV wt (*n* = 10, data not shown).

### 3.3. Growth Kinetics of Parental and Reassortant Viruses in Cell Lines from Diverse Host and Vector Species

**Figure 3:**
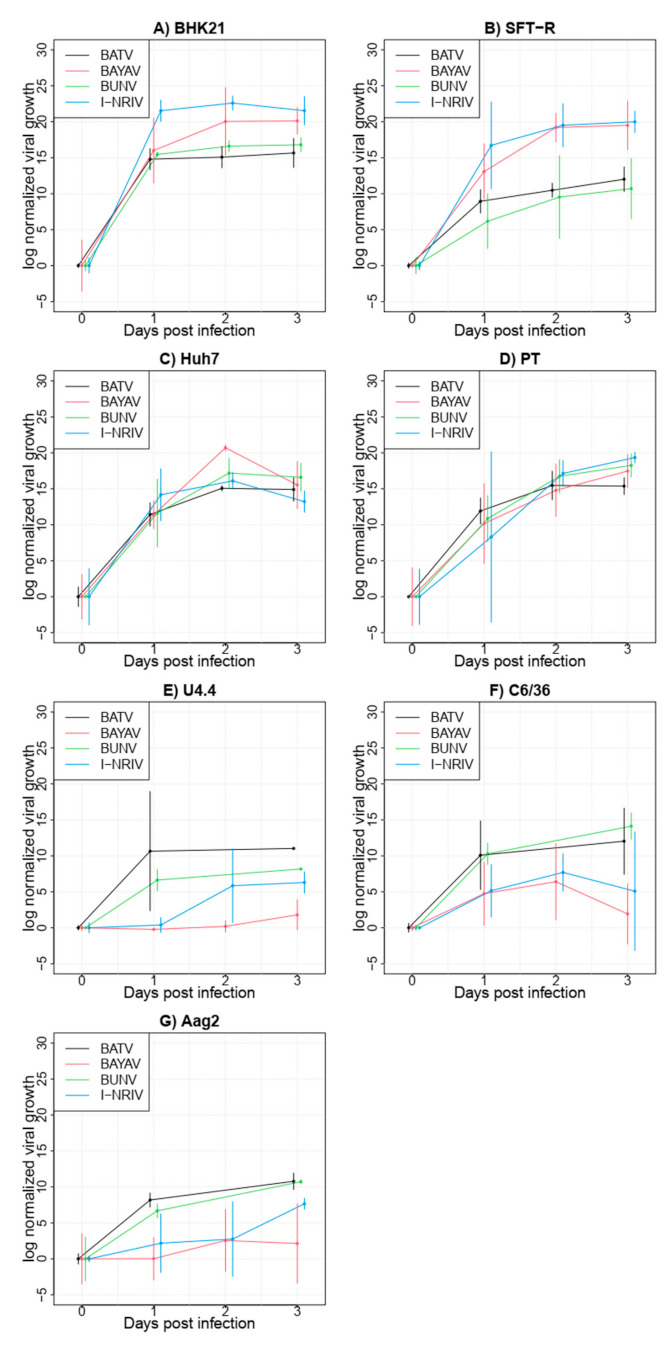
Replication kinetic in BHK-21 (**A**)**,** SFT-R (**B**)**,** Huh7 (**C**), PT (**D**)**,** U4.4 (**E**)**,** C6/36 (**F**) and Aag2 (**G**) cells. All cell lines were infected with an MOI of 0.1 and incubated for the indicated time. Each replication kinetic was repeated three times.

Next, we compared the growth curves of two clones of the newly emerged I-NRIV and BAYAV to the parental viruses BUNV and BATV. Therefore, we infected a panel of mammalian and insect cells and harvested supernatants 0, 24, 48 (only mammalian cells) and 72 h post-infection. First, values were normalised to eliminate measured virus contents at time point zero by calculating the mean for each virus at time point zero and dividing all values for this virus by this mean. Next, these ratios were log-transformed for variance stabilisation. For visualisation, time-specific 95% confidence intervals for each virus of the form “mean +/− z1-α/2 * standard error of the mean” were calculated, where z1-α/2 denotes the 1-α/2-quantile of the standard normal distribution (Figure 3). Raw data are presented in Supplementary Table S2. All viruses showed considerable growth in each of the tested cell lines (Figure 3). High variations as expressed by the standard error of the mean indicated considerable variances between different experiments. Despite this variance, the growth curves of BAYAV and I-NRIV were higher in BHK-21 (Figure 3A) and SFT-R cells (Figure 3B) but not in Huh7 (Figure 3C) and PT cells (Figure 3D). In contrast, the growth curves of BAYAV and I-NRIV were lower in insect cells compared to the parental BATV and BUNV (Figure 3E U4.4, Figure 3F C6/36 and Figure 3G Aag2).

Time curves in each cell line were analysed by a two-way analysis of variance, including the effect of the four viruses, the effect of the three/four time points and the interaction between viruses and time. Studying the interaction is of particular interest to detect whether time profiles of individual viruses are parallel and differ from each other. The variance analysis confirmed the growth of all viruses in all cell lines shown by the significant effect of the variable time (p_time_, Table 1). In contrast, the variable virus did not yield a significant effect (p_virus_, Table 2). However, when combining the time and virus, e.g., comparing the growth curves of those viruses, we found a significant difference in Aag2 cells and growth curve differences in C6/36 and SFT-R cells were close to significance in this full model (Table 1).

Next, we compared pairs of viruses (BATV vs. BAYAV; BATV vs. I-NRIV; BUNV vs. BAYAV; and BUNV vs. I-NRIV) in the three cell lines with significant or close to significant variation in growth curves in the full model (Table 2). In this reduced model, we found no significant difference between I-NRIV and BATV and BUNV in all three cell lines, whereas BAYAV showed close to significant different growth compared to BUNV in all three cell lines and compared to BATV in Aag2 and SFT-R cells.

## 4. Discussion

It is beneficial for each virus to replicate its genome to the highest possible number during infection. We observed a higher proportion of BUNV isolates in our co-infection studies after co-infections in BHK-21 cells (*Mesocricetus auratus*) and U4.4 and C6/36 cells (*Ae. albopictus*), indicating a higher fitness of BUNV in the tested cell lines. However, replication rates of BUNV and BATV were very similar in single infections in those cell lines (Figure 3A,E,F). Furthermore, other studies showed no significant differences in the growth kinetics among BATV, BUNV and NRIV in experiments with Vero cells (*Chlorocebus aethiops*) [22]. The presumed higher fitness of BUNV in the three tested cell lines might be explained by the protein interaction with BATV, which negatively influences BATV replication in the co-infection. Alternatively, BUNV replication rates in these cell lines in a co-infection might be higher than BATV genome replication. The co-infection interference of orthobunyaviruses has been described in mammals and insects, although the mechanisms behind this interference are not revealed [23,24]. Further experiments would be needed to analyse this hypothesis in more detail.

The possibility for reassortment of *Orthobunyaviruses* has already been demonstrated experimentally and in nature [8,11,13,25]. Our studies show that efficient reassortment was only observed in the 50:1 (BATV: BUNV) co-infection in BHK-21 cells. Furthermore, we observed reassortment via diploid viruses. The observed higher proportions of diploid viruses in the 50:1 ratio might argue that BATV genome replication is hindered by BUNV co-infection. However, other reasons, such as the inefficient packaging of BATV genome segments, might contribute to the low frequency of diploid viruses.

Interestingly, we did not observe any reassortment in U4.4 cells, although this was the only cell line in which BATV wt were isolated after co-infection (6%). Thus, it seems unlikely that an increased MOI of BATV would improve the reassortment frequency in these cells. Indeed, all tested diploid viruses from U4.4 showed a reversion to the BUNV wt after the second plaque-purification. The co-infection experiments in immune-competent (U4.4) and immune-deficient (C6/36) insect cells showed that the proportion of BUNV isolates is significantly higher in C6/36 cells than in U4.4 cells. The growth rates during single infections for both viruses in U4.4 and C6/36 cells (Figure 3E,F) displayed a better growth of BATV in U4.4 cells but not in C6/36 cells or Aag2 cells, where both viruses showed similar growth curves. Many viruses can escape from antiviral small interfering (si) RNA pathways and other antiviral mechanisms. It has been shown that the NSs-protein of BUNV is essential for viral replication in U4.4 but not in C6/36 [26], indicating a function necessary to escape the siRNA pathway. Potentially, BATV NSs has a similar function during replication; however, a NSs deletion mutant of BATV would be needed to show this. Furthermore, the observed BATV wt clones in U4.4. cells suggest that BATV has higher fitness in these cells than in C6/36 cells. This fitness could be attributed to the fact that our BATV isolate was derived from a mosquito in 2011 and is still well adapted to the insect vector or interaction with the insect’s immune system.

As already mentioned, we observed many isolates that did not show the classical three viral segments. The first group, the diploid viruses, harboured one additional segment: the S-segment was most frequent, followed by M-segment and L-segment diploids. Diploid viruses have been described before [17,18] and are speculated to be an intermediate step for reassortment [18]. However, we observed no S-segment reassortants despite the high number of diploid isolates with two S-segments (example in Supplementary Figure S1B). On the other side, reassortments observed after a second plaque-purification of diploid viruses were only M- and L-segment reassortants. Thus, we cannot deduce with any certainty from the data presented if these diploid viruses are a random phenomenon without any consequences for reassortment or a natural step in reassortment. Besides diploid viruses, we observed some isolates with less or equal to two segments. Since we are not aware of any description of naturally occurring two-segmented bunyaviruses that can replicate (this being a prerequisite for their isolation via plaque-purification), we assumed that these isolates are incomplete isolates, or that incomplete PCR data might have resulted from inhibition in the PCR reaction or incorrect RNA isolation.

When comparing the growth kinetics of BATV, BUNV and the two reassortant viruses I-NRIV and BAYAV, the reassortant viruses showed higher replication rates in the mammalian cell lines BHK-21 and SFT-R. However, only the virus growth curve for SFT-R was significantly different between the four viruses (Table 1 and Table 2). BHK-21 cells were kidney fibroblasts derived from the Syrian gold hamster (*Mesocricetus auratus*), whereas SFT-R cells originated from the thymus of the domestic sheep (*Ovis aris*). The other two cell lines which showed no growth differences were the human hepatoma cell line Huh7 and PT cells derived from the kidney of the domestic sheep (*Ovis aris*). Huh7 and BHK-21 cells were both used to study the cell entry of the Uukuniemi virus (UUKV, *Phenuiviridae*, *Bunyavirales*), and BHK-21 seem to be more susceptible to UUKV than Huh7 cells. The ovine PT and SFT-R cells have not been used in comparative infections with bunyaviruses; however, growth of the Schmallenberg virus was demonstrated in SFT-R cells. SFT-R and PT cells were used in a comparative study testing the growth of different pestiviruses [27]. Both ovine cell lines, PT and SFT-R, could be infected with all tested viruses to the same extent, whereas Huh7 cells were only infected with one of the six tested viruses. Furthermore, infection experiments in the two ovine cell lines with the bluetongue virus (BTV), serotype 1 and 8 reassortants, revealed that the reassortment phenotype (growth behaviour similar to BTV8) was more pronounced in SFT-R as compared to PT cells; however, both BTV1 and BTV 8 grew to the same titer in both cell lines [28]. We observed very similar growth curves for BATV and BUNV in BHK-21 and Huh7 cells with identical endpoint virus titers (Figure 3, Table S2). However, BAYAV and I-NRIV grew to higher titers in BHK-21 cells, indicating a specific interaction of the reassortant viruses with both cell lines. A possible factor contributing to the observed differences could be the activation of interferon via the cellular receptor RIG-I. This DExD/H box RNA helicase activates interferon expression upon recognition of viral genomic RNA structures and has been demonstrated to be essential for recognising many bunyavirus infections [29]. Whereas RIG-I dependent activation of the interferon pathway is intact in Huh7 cells [30], severe impairment of RIG-I-dependent recognition of viral genomes has been demonstrated in BHK-21 cells [31]. This impairment might facilitate better growth of BAYAV and I-NRIV in BHK-21 cells. However, multiple factors can influence parental and reassortant virus growth in BHK-21 and Huh7 cells since these cell lines were derived from different species and different organs; thus, species or organ-specific differences might play a role. The PT and SFT-R cells were derived from the same species, the domestic sheep, which makes the results obtained in these cells more comparable. In contrast to Huh7 and BHK-21 cells, we observed similar titers of the reassortant viruses BAYAV and I-NRIV in both cell lines (Figure 3, Table S2), but the parental virus growth was lower in SFT-R cells compared to PT cells. SFT-R cells were shown to be interferon-competent, and the deletion of the NSs protein of the Schmallenberg virus had a severe impact on the growth of this mutant virus in those cells [26]. Unfortunately, nothing has been published on the interferon pathway in PT cells; thus, we can only speculate that the lower growth rates of BATV and BUNV in SFT-R cells are due to the interferon pathway-dependent inhibition of virus growth. However, a better inhibition of interferon induction can be one reason for improved viral growth (reviewed by the authors in [32]). Furthermore, a study by Tilson et al. [33] using minigenomes and virus-like particles to create diverse reassortants of *Oropouche orthobunyaviruses* and Schmallenberg virus showed that, for example, the combination of the Oropouche M minigenome with the L- and N-proteins of Schmallenberg virus led to a 50-fold increase in minigenome activity. In contrast, the other combinations did not show increased activity. These results indicate that some new combinations of genomic segments and proteins from different viruses can enhance the transcription activity, leading to higher replication rates. The effect could be cell-type specific and contribute to our reassortant viruses’ observed enhanced growth rates in some cell lines.

Taken together, the different growth kinetics of reassortant and parental viruses in the four tested mammalian cell lines might be correlated with the different interactions with the host immune system. However, tissue and host-specific interactions independent of immune system–virus interactions or viral transcription and replication changes could be responsible. More experiments targeting specific interaction with the immune activation pathway and the use of minigenome systems to determine virus replication and transcription could help to understand the nature of differences between reassortants and parental viruses.

The replication rates for the two reassortants were lower in insect cells, with differences close to significance for C6/36 and significant differences for Aag2 cells compared to the parental viruses. In the reduced model, where we compared the reassortant viruses with each parental virus, we observed a more substantial effect for BAYAV than I-NRIV in C6/36 and Aag2 cell lines. As already mentioned above, C6/36 cells have an impaired RNA interference pathway [21], which might lead to differences in viral growth. BAYAV carries the BATV S-segment and thus expresses the BATV NSs-protein, which might confer a similar phenotype as observed for BATV. However, since we did not observe growth differences in U4.4 but rather in C6/36, the role of NSs-Dicer-2 interaction for different growth kinetics might be neglected. The most noticeable effect of the reduced BAYAV growth was observed in the *Aedes aegypti* Aag2 cells, indicating a species-specific effect. Along this line, a study using *Aedes aegypti* mosquitos demonstrated that these mosquitos were moderately susceptible to BUNV, whereas the same mosquito strain was refractory to NRIV [34]. Taken together, the potential impairment of reassortant bunyaviruses in *Aedes aegypti* could be suspected.

The nature of this impairment is still enigmatic; Aag2 cells have an intact Dicer-2 and PIWI pathway [21], making RNA interference pathway impairments unlikely to be responsible for the observed phenotype. Persistent infection with insect-specific viruses could account for different virus growth kinetics. It has been shown that the cell fusing agent virus (CFAV), which persistently infects Aag2 cells, influences the growth of the dengue virus in this cell line [35]. With regards to our cells used in the experiment, we confirmed CFAV in our Aag2 cells. Perhaps CFAV could play a role in suppressing BAYAV and I-NRIV; however, more in-depth sequencing is needed to analyse if CFAV may play a role in growth differences.

Interestingly, we neither observed reassortment in all tested insect cells nor enhanced growth of reassortant I-NRIV and BAYAV. This observation contradicts current literature, which describes reassortment primarily in the insect vector but not in mammalian hosts. Thus far, studies to demonstrate genome reassortment in vertebrates have been unsuccessful [12], while the reassortment of heterologous and homologous orthobunyaviruses was demonstrated in the mosquito vector [36,37]. However, a reassortment in mammalian hosts in nature cannot be ruled out since *Orthobunyavirus* reassortants such as the Ngari virus have been isolated from both insect and mammalian hosts [8,38]. The observed differences between some insect vector cells (specifically Aag2) and some mammalian cell lines (BHK-21 and SFT-R) with the same novel combination of segments might point to a beneficial role of the novel combination in one system, which is disadvantageous in another system and can lead to a different adaptation of these new viruses to hosts and vectors. To further investigate the role of protein combination and adaptation processes, a reverse genetic system for BATV would be helpful. With the help of such reverse genetic systems, new reassortants could be generated using the already established BUNV reverse genetic system [39], so the impact of diverse segment combinations could be tested.

## 5. Conclusions

In conclusion, we could reconstruct a natural reassortant of BATV and BUNV, the NRIV, in an experimental co-infection setting. Furthermore, we obtained a novel reassortant virus in this system, provisionally named BAYAV, which harbours a novel segment composition that has not been described before. We observed reassortment of those two viruses solely in mammalian BHK-21 cells, which contradicts previous studies on orthobunyavirus reassortment, stating that reassortment processes take place in the insect host. Both reassortants showed enhanced growth in some mammalian cells and were impaired in all insect cells, indicating a specific adaptation to the mammalian cell lines BHK-21 and SFT-R through reassortment. The nature of this adaptation is not yet clear and warrants further investigation.

## Figures and Tables

**Table 1:** Results from the full model, including all four viruses. Significant *p*-values are marked in bold.

Tissue	p_virus_	F-Value	p_time_	F-value	p_virus × time_	F-Value
Aag2	0.6165	(0.6)	<0.0001	(54.9)	**0.0500**	(3)
BHK-21	0.9015	(0.2)	<0.0001	(75.8)	0.6741	(0.5)
C6/36	0.9939	(0)	<0.0001	(27.9)	0.0625	(2.8)
Huh7	0.9515	(0.1)	<0.0001	(74.2)	0.8004	(0.3)
PT	0.9154	(0.2)	<0.0001	(152.7)	0.6359	(0.6)
SFT-R	0.6722	(0.5)	<0.0001	(106.9)	0.0554	(2.8)
U4.4	0.2571	(1.4)	<0.0001	(33.3)	0.1012	(2.3)

**Table 2:** Reduced model comparing all four viruses in selected cell lines. Due to four subgroup analyses following non-significant results in the full model, a *p*-value smaller than a Bonferroni-corrected significance level of 0.05/4 = 0.0125 is considered significant.

Reduced Model	Tissue	p_virus_	F-Value	p_time_	F-Value	p_virus × time_	F-Value
BATV versus BAYAV	Aag2	0.3583	(0.9)	0.0013	(16.4)	0.0374	(5.3)
	C6/36	0.8884	(0)	0.0091	(9.4)	0.066	(4)
	SFT-R	0.6625	(0.2)	<0.0001	(68.1)	0.0409	(4.8)
BATV versus I-NRIV	Aag2	0.2534	(1.4)	<0.0001	(36.9)	0.3426	(1)
	C6/36	0.8103	(0.1)	0.0028	(13.5)	0.2387	(1.5)
	SFT-R	0.4093	(0.7)	<0.0001	(42.8)	0.116	(2.7)
BUNV versus BAYAV	Aag2	0.5089	(0.5)	0.0006	(19.6)	0.0232	(6.6)
	C6/36	0.9300	(0)	0.0019	(14.6)	0.0176	(7.3)
	SFT-R	0.5290	(0.4)	<0.0001	(79)	0.0168	(6.7)
BUNV versus I-NRIV	Aag2	0.3900	(0.8)	<0.0001	(45.3)	0.2613	(1.4)
	C6/36	0.8363	(0)	<0.0001	(19.8)	0.0861	(3.4)
	SFT-R	0.3125	(1.1)	<0.0001	(49.6)	0.0632	(3.8)

## Data Availability

The data presented in this study are available on request from the corresponding author.

## Supplementary Materials

Supplementary materials can be found at https://www.mdpi.com/article/10.3390/v13091702/s1. Table S1: Primer used for RT-PCR analysis of reassortant virus clones from co-infection experiments; Table S2: Viral growth kinetic in mammalian and insect cell lines; Figure S1: Pie chats representing the fraction of BUNV, BATV, Diploid viruses and reassortant viruses and viruses with less than 3 segments (N/A) from total number of analysed clones in the 1:1 infection.
