# Supplementary material for: Mammals Preferred: Reassortment of Batai and Bunyamwera orthobunyavirus Occurs in Mammalian but Not Insect Cells
Source: Viruses. 2021 Aug 27;13(9):1702. doi: 10.3390/v13091702 (PMC8473249; doi:10.3390/v13091702)
Supplement: Supplementary file 1 [file viruses-13-01702-s001.zip › viruses-1313991-supplementary.pdf]

Supplementary Table S1: Primer used for RT-PCR analysis of reassortant virus clones from co-infection experiments.

Virus & Segment	Fragment length [bp]	Primer sequence
BATV L-Segment	710	5'-GATGGCGATTTCCTGATTAT-3' †
		3'-TGACCCCAAGAGTTTCCTATTAT-5' †
BATV M-Segment	875	5'-TGGCCTAGCATATCACCCTTTCAC-3'
		3'-GTTCTTTGCCTTCTTATGCTTGCTATTG-5'
BATV S-Segment	633	5'-TGCTAACACCAGCAGTACTT-3'
		3'-AGCACTGCTTACATTTGTCT-5'
BUNV L-Segment	1038	5'-GACAGTATAATTAAAGGAGACC-3'
		3'-GCTTATCAATTGAATTGCATAAG-5'
BUNV M-Segment	745	5'-GAAGAAGACTGCTGGAAAAAT-3'
		3'-CCACCAGATCGGGTTAATTC-5'
BUNV S-Segment	771	5'-AATCGCTGTGCTATTAAATCC-3'
		3'-CCCGATTAAAAATGCATCCC-5'

† BATV L-Segment primer based on Jöst et al. (2011)

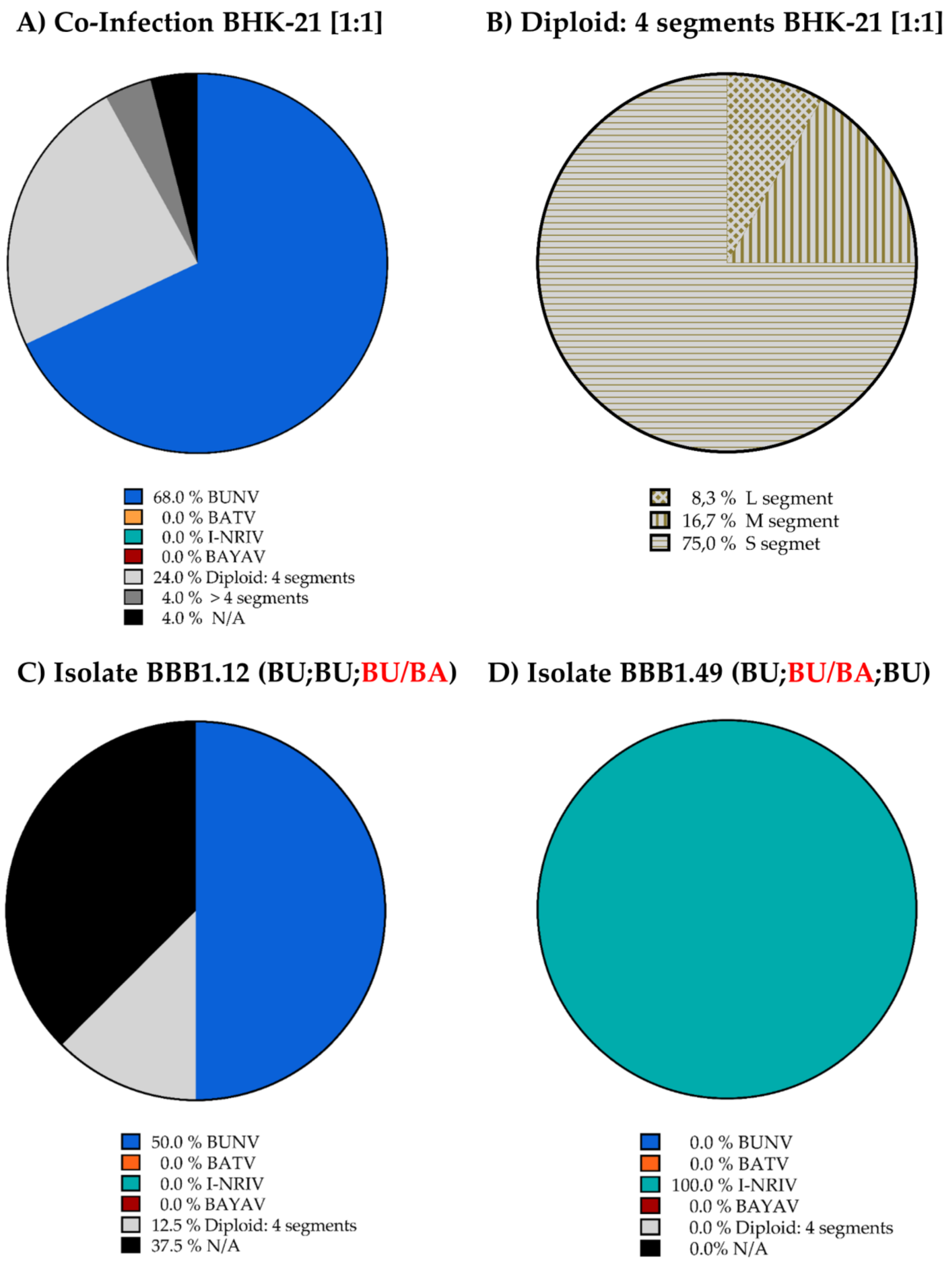

Figure S1: Pie chats representing the fraction of BUNV, BATV, Diploid viruses and reassortant viruses and viruses with less than 3 segments (N/A) from total number of analysed clones in the 1:1 infection. **(A)** results of the 1:1 BATV: BUNV infection in BHK-21 cells total n=50 **(B)** separated proportions of L, M and S-segment diploid viruses from the fraction diploid viruses in panel (A) n=12 **(C)** results of the second plaque purification performed with the clone BBB1.12 (S-segment diploid virus) n=8 and **(D)** results of the second plaque purification of the M-segment diploid virus clone BBB1.49

Supplementary Table 2: Viral growth kinetic in mammalian and insect cell lines. All values originate from three independent infection experiments of BATV, BUNV, I-NRIV and BAYAV in the named cell lines. The experiment number is given as kinetic # , Samples were taken just after infection (=T0) and 24 (=T1), 48 (=T2) and 72 (=T3) hours post infection. All values are given as plaque forming units per mL.

cell line	virus	kinetic #	T0	T1	T2	T3
Aag2	I-NRIV	1	1,0E+00	1,0E+00	1,9E+00	3,9E+03
		2	1,9E+00	6,9E+02	3,9E+03	3,9E+03
		3	1,0E+00	1,9E+00	1,0E+00	1,2E+03
	BAYAV	1	2,2E+02	1,2E+02	1,2E+03	1,1E+01
		2	1,0E+00	1,0E+00	1,0E+00	1,0E+00
		3	1,0E+00	1,9E+00	3,9E+02	1,2E+04
	BATV	1	1,5E+00	2,9E+03		9,2E+04
		2	3,5E+00	1,6E+04		2,2E+05
		3	0,0E+00	5,2E+03		2,9E+04
	BUNV	1	8,2E+00	2,9E+04		5,2E+05
		2	1,5E+00	5,2E+03		6,9E+05
		3	2,9E+02	1,2E+04		9,2E+05
C6/36	I-NRIV	1	1,0E+00	6,1E+00	2,2E+03	1,0E+00
		2	1,0E+00	3,9E+03	2,2E+04	6,9E+05
		3	1,0E+00	2,2E+02	2,2E+02	6,1E+00
	BAYAV	1	1,0E+00	3,9E+02	6,9E+03	1,0E+00
		2	1,9E+00	3,9E+03	1,8E+04	6,1E+02
		3	1,0E+00	1,9E+00	3,5E+00	1,0E+00
	BATV	1	9,2E+02	1,2E+06		1,2E+08
		2	2,9E+02	2,2E+06		6,9E+07
		3	5,2E+02	3,9E+06		6,9E+07
	BUNV	1	2,6E+00	6,9E+04		6,9E+06
		2	4,6E+00	3,9E+05		6,9E+05
		3	2,6E+00	2,9E+04		1,6E+07
U4.4	I-NRIV	1	1,0E+00	1,0E+00	6,6E+03	2,2E+03
		2	3,5E+00	6,1E+00	1,2E+04	2,2E+03
		3	1,9E+00	3,5E+00	3,5E+00	2,2E+02
	BAYAV	1	1,9E+00	1,0E+00	3,5E+00	1,0E+00
		2	1,0E+00	1,0E+00	1,0E+00	3,9E+01
		3	1,0E+00	1,0E+00	1,0E+00	1,1E+01
	BATV	1	0,0E+00	2,9E+02		6,9E+04
		2	1,5E+00	9,2E+06		8,2E+04
		3				

	**BUNV**	1	8,2E+00	2,9E+03		2,9E+04
		2	8,2E+00	2,9E+04		2,9E+04
		3	8,2E+00	2,9E+03		2,9E+04
**BHK21**	**I-NRIV**	1	3,5E+00	1,6E+09	2,2E+10	5,2E+09
		2	6,1E+00	2,2E+10	6,9E+09	1,2E+09
		3	0,0E+00	6,9E+09	1,2E+09	3,9E+10
	**BAYAV**	1	2,6E+00	2,2E+07	2,2E+10	2,9E+10
		2	5,2E+02	1,2E+10	2,2E+11	1,2E+09
		3	1,9E+00	6,9E+06	6,9E+07	1,2E+10
	**BATV**	1	1,9E+00	1,2E+07	2,2E+07	3,9E+07
		2	1,1E+00	6,9E+06	1,2E+06	2,2E+07
		3	1,1E+00	3,9E+05	1,2E+06	3,9E+05
		4	1,1E+00	3,9E+06	1,2E+07	1,2E+07
	**BUNV**	1	1,1E+00	6,9E+06	3,9E+07	1,2E+07
		2	5,3E+00	6,9E+06	3,9E+07	3,9E+07
		3	1,1E+00	1,2E+07	1,2E+07	6,9E+07
**Huh7**	**I-NRIV**	1	4,6E+00	1,2E+07	9,2E+08	5,2E+06
		2	1,6E+03	1,6E+09	1,2E+08	1,2E+07
		3	3,5E+00	3,9E+06	2,2E+08	6,9E+07
	**BAYAV**	1	8,2E+00	6,9E+05	2,2E+10	6,9E+06
		2	3,9E+02	5,2E+05	1,2E+10	2,2E+09
		3	1,9E+00	1,2E+07	2,2E+10	6,9E+07
	**BATV**	1	1,9E+00	2,2E+05	1,2E+07	3,9E+07
		2	1,1E+01	1,2E+06	6,9E+06	2,2E+06
		3	1,1E+00	6,9E+04	1,2E+07	6,9E+06
	**BUNV**	1	1,1E+00	3,9E+05	6,9E+07	1,2E+08
		2	1,1E+00	3,9E+06	1,2E+08	3,9E+06
		3	1,1E+00	1,2E+03	3,9E+06	1,2E+07
**PT**	**I-NRIV**	1	2,2E+00	1,8E+04	1,6E+09	3,9E+09
		2	5,2E+02	2,2E+09	2,2E+08	3,9E+09
		3	0,0E+00	1,9E+00	6,9E+07	1,2E+09
	**BAYAV**	1	4,6E+00	1,6E+04	9,2E+07	9,2E+08
		2	9,2E+02	1,2E+08	6,9E+08	3,9E+09
		3	1,0E+00	3,9E+04	1,2E+06	6,9E+07
	**BATV**	1	1,1E+00	9,2E+05	3,9E+07	1,6E+07
		2	1,1E+00	3,9E+04	3,9E+06	3,9E+06
		3	1,1E+00	1,2E+05	1,2E+06	2,2E+06
	**BUNV**	1	1,1E+00	3,9E+05	1,2E+08	9,2E+07
		2	1,1E+00	2,2E+05	3,2E+07	3,9E+08
		3	1,1E+00	2,2E+03	2,2E+06	2,2E+07
**SFT-R**	**I-NRIV**	1	4,6E+00	3,9E+07	2,2E+10	5,2E+09

	2	4,6E+00	1,6E+10	2,2E+08	3,9E+08
	3	1,9E+00	3,9E+05	2,2E+08	2,2E+09
**BAYAV**	1	2,6E+00	9,2E+06	1,6E+09	3,9E+09
	2	2,6E+00	6,9E+06	1,2E+09	3,9E+09
	3	1,9E+00	2,2E+04	6,9E+07	2,2E+07
**BATV**	1	1,9E+00	3,9E+04	3,9E+04	1,2E+05
	2	1,1E+00	1,2E+04	2,2E+04	6,9E+04
	3	1,1E+00	2,2E+03	1,2E+05	1,2E+06
**BUNV**	1	1,1E+00	2,2E+04	3,9E+06	2,2E+07
	2	1,1E+00	6,1E+00	3,9E+03	3,9E+03
	3	1,9E+01	1,2E+05	3,9E+07	1,2E+07
	4	1,1E+00	1,9E+01	1,9E+00	1,2E+03
	5	1,1E+00	2,2E+03	1,2E+04	3,9E+03
